# Behavior, behavioral syndromes, and metabolism: the effects of artificial selection for death-feigning on metabolic rate

**DOI:** 10.1093/jisesa/ieaf007

**Published:** 2025-02-15

**Authors:** Kentarou Matsumura, David J Hosken, Tomohito Noda, Takahisa Miyatake, Manmohan D Sharma

**Affiliations:** Graduate School of Environmental and Life Science, Okayama University, Okayama, Japan; Centre for Ecology and Conservation, Faculty of Environment, Science and Economy, University of Exeter, Penryn, UK; Graduate School of Arts and Sciences, The University of Tokyo, Komaba,Tokyo, Japan; Centre for Ecology and Conservation, Faculty of Environment, Science and Economy, University of Exeter, Penryn, UK; Centre for Ecology and Conservation, Faculty of Environment, Science and Economy, University of Exeter, Penryn, UK; Department of Biological Sciences, Graduate School of Science, The University of Tokyo, Tokyo, Japan; Bioproduction Research Institute, National Institute of Advanced Industrial Science and Technology (AIST), Tsukuba, Japan; Graduate School of Environmental and Life Science, Okayama University, Okayama, Japan; Centre for Ecology and Conservation, Faculty of Environment, Science and Economy, University of Exeter, Penryn, UK

**Keywords:** anti-predator behavior, artificial selection, death-feigning, metabolic rate, personality, *Tribolium*

## Abstract

Death-feigning, or thanatosis, is an anti-predator behavioral strategy in many animals. Because individuals remain immobile while feigning death, individuals with longer durations of death feigning often show lower locomotor activity. Thus, metabolic rate, which is closely related to locomotor activity, may also be related to the intensity of death feigning. If there is a genetic correlation between death feigning and metabolism, metabolic rate may respond to selection on death-feigning behavior. Here, we tested for a relationship between metabolic rate and death-feigning using replicated populations of the red flour beetle (*Tribolium castaneum*) subjected to artificial bidirectional selection on the duration of death-feigning behavior. The results indicated that metabolic rate did not differ between populations selected for increased or decreased death feigning, although locomotor activity was significantly different between these treatments; populations selected for reduced death-feigning durations tended to be more active. These results suggest that death-feigning behavior is not genetically correlated with metabolic rate in *T. castaneum*.

**Significance Statement**

Death-feigning behavior is frequently correlated with locomotor activity. Therefore, the intensity of death feigning may also be associated with energy consumption. Here, we compared the locomotor activity and metabolic rate between two populations that were artificially selected for longer or shorter durations of death feigning in the red flour beetle *Tribolium castaneum*. The results showed that although the duration of death feigning was negatively correlated with locomotor activity, there was no correlation between death feigning and metabolic rate. These results suggest that death-feigning behavior is not genetically correlated with metabolic rate in *T. castaneum*.

## Introduction

Predation is an important selection pressure for prey species (eg Lima and Dill 1990, Abrams 2000, Caro 2005), and as a result, prey have evolved a range of anti-predator behaviors to facilitate escape in encounters with predators (eg Ohno and Miyatake 2007, Humphreys and Ruxton 2018). Thanatosis, or death-feigning, is one such anti-predator behavior which is found in many species, and numerous studies have documented its utility in predation avoidance (eg Miyatake et al. 2004, 2009, Honma et al. 2006, Hansen et al. 2008). Interestingly, there is considerable intra-specific variation in the duration of death-feigning (Humphreys and Ruxton 2018). This variation could be maintained by consistent combinations of behaviors (behavioral syndromes), or perhaps there are costs of death feigning in other situations. Consistent with this latter suggestion, in the adzuki bean beetle (*Callosobruchus chinensis*) and red flour beetle (*Tribolium castaneum*), individuals with longer duration of death feigning had decreased mating success (Nakayama and Miyatake 2010, Nakayama et al. 2010). Furthermore, because individuals with longer duration of death feigning had significantly lower locomotor activity (eg Miyatake et al. 2008, Matsumura et al. 2017), it was assumed that they experienced decreased encounter rates with potential mates than individuals with shorter duration of death feigning (Nakayama and Miyatake 2010, Nakayama et al. 2010). Therefore, death feigning can be costly (in non-predation situations), and this could maintain variation in its duration.

Locomotor variation (and variation in other activity levels) is not only correlated with death feigning but has also been linked to variation in metabolic rate (MR) (e.g., Crnokrak and Roff 2002, Okada et al. 2011. And see review in, Biro and Stamps 2010). Metabolic rate is often measured as basal or resting metabolic rate (RMR) and is an indicator of the general resources required for somatic maintenance, and RMR frequently correlates with body size and life history (e.g., Sinclair et al. 2010, Okada et al. 2011, reviewed in McNab 2002). Because of its central role in resource allocation (Brown et al. 2004, McGill et al. 2006, Burton et al. 2011, Okada et al. 2011), behavioral ecologists have become increasingly interested in metabolism and the possible role it may play in generating variation in behavioral traits and personality (behavioral syndromes) within populations (reviewed in Réale et al. 2007, 2010, Biro and Stamps 2010). For example, in the sand cricket *Gryllus firmus*, larger wing-morph individuals, which have greater dispersal ability, have higher MR than smaller wing-morph crickets (Crnokrak and Roff 2002). Metabolic rate and personality are predicted to be linked through pace-of-life associations (Réale et al. 2010)—relatively shy, less-active individuals may have lower MR for example, or perhaps individuals with higher MR may have to take more risks to fuel their higher MR (White et al. 2016). Furthermore, under different models of activity- MR association that have been expanded to encompass personality, MR and activity can be positively or negatively associated (performance vs allocation model predictions respectively: for details of these models see Careau et al. 2008). However, clear behavioral syndrome-MR links are relatively rare (Careau et al. 2008, White et al. 2016). Nonetheless, and as noted above, if different combinations of MR and personality attributes (or single behaviors) return equal fitness, variation in character combinations, including death feigning and activity, could be maintained (Biro and Stamps 2010; Réale et al. 2010).

Given the associations between death feigning and locomotion, and locomotion and RMR, it is possible that death-feigning behavior linked with locomotor activity may also be correlated with MR. This has been documented in the beetle *Tenebrio molitor* where the duration of tonic immobility (ie death-feigning) is negatively phenotypically associated with RMR (Krams et al. 2013). While that study did not test for links with general activity levels, individuals that feign death more often had lower RMR than individuals who tended to flee from predators. Similarly, in great tits *Parus major*, the time to resume feeding post-disturbance can be negatively associated with MR (depending on the disturbance type) (Mathot et al. 2014). In contrast, a previous study reported that walking activity of *T. castaneum* was not correlated with MR (Arnold et al. 2017). Therefore, relationships between death feigning, locomotor activity, and MR remain somewhat unclear and may simply differ across species (Krams et al. 2013, Arnold et al. 2017).

Artificial selection to shift focal traits and increase phenotypic variance is a powerful method to investigate trait associations (Falconer and Mackay 1996). Many previous studies have used this approach to investigate trait correlations (e.g.,van Dijken and Scharloo 1979a, b, Wilkinson and Reillo 1994, Swallow et al. 1998, Koch and Britton 2001, Miyatake et al. 2004), including associations between activity levels and other characteristics (eg Matsumura et al. 2019, Uchiyama et al. 2019). For example, in house mice *Mus domesticus*, artificial selection for locomotor activity has been shown to alter body weight at maturity (Swallow et al. 1999). Artificial selection for *Drosophila melanogaster* locomotor activity has also been shown to alter reproductive traits (van Dijken and Scharloo 1979b) . Additionally, this approach is useful for investigating the genetic basis of behavioral syndrome, as seen in studies of *P. major* (Drent et al. 2003, van Oers et al. 2004).

Here, we examined the potential relationship between death-feigning behavior and metabolic rate in the red flour beetle *T. castaneum*. Replicate populations of *T. castaneum* were subjected to bidirectional artificial selection on the duration of death-feigning behavior for > 40 generations, to establish populations of genetically longer and shorter durations of death-feigning (Miyatake et al. 2004, Matsumura and Miyatake 2018). If the pattern reported for *T. molitor* (Krams et al. 2013) is general, beetles from long-duration thanatosis populations should show significantly lower RMR than beetles from short-duration populations. Since *T. castaneum* and the *T. molitor* are closely related, this relationship may also be seen in *T. castaneum*. Alternatively, it is also possible that death feigning is not correlated with RMR because previous work (Arnold et al. 2017) found no relationship between MR and activity in *T. castaneum*, although this previous study did not focus on death feigning per se. To test these possibilities, we compared the RMR of beetles from populations with long and short durations of death-feigning as adults and pupae. Pupae were measured because metabolic rate can vary through ontogeny (eg Wieser 1984, Post and Lee 1996) and it is not clear when potential differences could manifest.

## Materials and Methods

### Insect and Artificial Selection

All populations of *T. castaneum* used in the present study were reared in incubators (Sanyo, Tokyo, Japan) maintained at 25 °C, approximately 60% r.h. and 16L8D (light on at 7:00, light off at 23:00) photoperiod with mixture of whole wheat flour (Nisshin Seifun, Tokyo, Japan) enriched beer’s yeast (Asahi Beer, Tokyo, Japan) as food. The stock culture was maintained by randomly pairing 10 virgin males and 10 virgin females to propagate the following generation. The insects were collected, sexed, and separated by sex during their pupal stage to ensure there were no prior matings, especially interbreeding between generations. The pupae were sexed based on pupal abdomen morphology and placed in petri dishes for emergence and maintained in sexed groups until use.

Beetles have been artificially selected for the duration of death feigning for over 15 yr to establish a long-duration population (L-population) and a short-duration population (S-population) (see Matsumura and Miyatake 2018). To initially establish the populations, pupae were randomly collected from the stock culture, sexed, and placed in separate, sex-specific, petri dishes for emergence and maintained in sexed groups till pairing. One hundred virgin males (14–21 d old) and 100 virgin females (14–21 d old) were placed into individual wells of 48-well plates with food. During the next 136 d, duration of death feigning of each beetle was measured (see below). The 10 males and 10 females with the longest duration of death-feigning were collected and used to propagate the L-population, whereas the 10 males and 10 females with the shortest duration of death feigning were collected and used to propagate the S-population. The offspring of each population/treatment were reared in environment controlled growth chambers as described above. Pupae emerged about 40 d after hatching, and the pupae were separated by sex until emergence. When the next generation of adults emerged, we measured the duration of death feigning and selected those with the longest and shortest death-feigning durations in the same manner as before. We simultaneously created 2 replicates per population (ie LA, LB, SA, and SB). We repeated this procedure for over 40 generations (see Matsumura and Miyatake 2018). All trials in the present study were conducted between 12:00 and 17:00 in a room maintained at 25°C.

### Death Feigning

One hundred virgin males and 100 virgin females (21 to 35 d post- eclosion) were randomly collected from each population and were placed alone in individual wells of 48-well tissue culture plates (Falcon; Becton– Dickinson and Co., Franklin Lakes, NJ, USA) with food. To examine the duration of death feigning, a beetle was gently moved onto a small white China saucer (140 mm diameter, 15 mm deep). Death feigning was induced by touching the abdomen of the beetle with a wooden stick. A trial consisted of provoking the death feigning and recording its duration. The duration of the behavior was defined as the length of time between touching the beetle and detecting its first visible subsequent movement. If the beetle did not become immobile, the touch was repeated up to 3 times. If the beetle was unresponsive to stimulation, its death-feigning duration was recorded as zero.

### Locomotor Activity

Virgin males and virgin females (21 to 35 d post-eclosion) were randomly collected from L- (male: *N* = 48; female: *N* = 65) and S-populations (male: *N* = 47; female: *N* = 70), and their locomotor activity was measured for 24h. To measure locomotor activity, we used an infrared actograph (E3S-AT11; Omron, Kyoto, Japan). In this monitoring system, an infrared light beam was passed through a Petri dish with a beetle without food. When the beetle interrupted the light beam, a signal is recorded immediately, and locomotor activity was defined as the number of interruptions of the light beam in 24 h (Supplementary Fig. S1). Each individual was placed in a transparent plastic Petri dish (30 × 10 mm) lined with filter paper at the base to prevent the beetle from slipping within the smooth container. Measurements were conducted in an incubator maintained at 25 °C in darkness. To remove the effect of placing the beetles in the container, measurements were started 6 h after the beetles were placed.

### Metabolic Rate

We measured the RMR of beetles from L- and S-populations at pupal and adult stages in a flow-through system (Sable Systems, Las Vegas, USA) (Lighton 2008). At pupation, we measured body mass (*N* = 120 and *N* = 118 for the long- and 181 short-duration treatment) and CO_2_ production as our measure of RMR (see methods in Arnqvist et al. 2010, Sinclair et al. 2010, Okada et al. 2011). Briefly, compressed zero-air (79% N_2_ and 21% O_2_) was first scrubbed of residual CO_2_ and water (Ascarite II and Magnesium Perchlorate) and regulated at a flow rate of 100ml min-1 ± 1% with a Sierra Instruments (Monterey, CA, USA) model 840L mass-flow meter connected to a mass-flow control unit (Sable Systems Intelligent Mass Flow Control Unit, MFC-2, Las Vegas, USA). Air was then fed into a recently calibrated LI-COR LI- 7000 infra-red gas analyzer (IRGA) capable of resolving differences of 1ppm of CO_2_ in the air, plumbed in differential mode (see Lighton 2008). Air exiting the IRGA reference cell was passed through a Sable Systems (Las Vegas USA) RM8 eight-chamber multiplexer and then forwarded to the sample cell for measurements. A PC running Sable Systems Expedata software (ver.1.8.2) and connected to a Sable Systems UI2 analog–digital interface was used for data acquisition. Within the multiplexer, one empty chamber acted as a negative control and beetles were haphazardly assigned to other chambers to minimize positional bias. Baseline measurements were made before and after each set of recordings to test and control for any drift between calibrations.

Data (Sharma, Rogerson et al. unpublished) collected across a range of test periods (up to 30 min) were used to identify a suitable measurement period that yielded a stable RMR while minimizing the potential for stress-induced changes in desiccated air using both open and closed respirometry systems. A flow-through respirometry system with at least 10 min prior to measurement and a 7-min measurement phase was selected to provide the most stable measurement duration (also see Woods 208 and Hill 2004, Lehmann et al. 2014). VCO_2_ was measured for 7 min/beetle (with a sample read every second), and data from the most stable section (Lighton 2008 and Lighton and Halsey 2011 ) of the last 2 min of measurement (to control for wash-out) were used to calculate the average CO_2_ production for each individual (ranging from 60 to 100 s of measurement/beetle). The stable section was assessed manually using the Expedata software (ver.1.8.2).

For pupae, after measurement of metabolic rate, individuals were placed into 24-well plate separately with food and maintained until they became adults. The RMR of a subsample of virgin males (*N* = 50 and *N* = 51 for long- and short-duration treatments) and females (*N* = 55 and *N* = 54) was measured again at adulthood using the methods described above. To avoid the effect of feeding, adult beetles (10 to 15 d old) were isolated without food for 1 h before the measurement of metabolic rate. All measurements of RMR were conducted between 12:00 and 18:00 in an incubator maintained at 25 °C. We additionally conducted a repeatability assessment of our metabolic rate assessment in adults (not in pupae) as motility in adults, as opposed to pupae, is apparent. We measured 6 individual adults 3 times within a 24-h period and calculated the repeatability of RMR across these measurements (Lessells and Boag 1987), which was *r* = 0.99. Thus, our measurement of adult MR was highly repeatable.

### Statistical Analysis

To test for effects of direct responses to artificial selection for duration of death feigning, we used a generalized liner mixed model (GLMM), with gamma distribution (link: log), population (L and S) and sex as fixed effects, and replicate line as random effects. To test for effects of indirect responses to artificial selection for duration of death feigning on locomotor activity, we used GLMM with gamma distribution (link: inverse), population (L and S) and sex as fixed effects, and replicate line (A and B) as random effects. Body mass at pupal and adult stages were analyzed by GLMM with Gaussian distribution (link: log), population and sex as fixed effects and replicate line as a random effect. To test for population effects on metabolic rate at pupal and adult stages, we used GLMM with gamma distribution (link: identity for pupa, inverse for adult), body mass as a covariate, population, and sex as fixed effects and replicate line as random effects. To test for association between pupal and adult stages in metabolic rate and body mass, we used linear regression analysis. All analyses were conducted by R version 4.1.0 (R Core Team 2021).

## Results

**Fig. 1.**
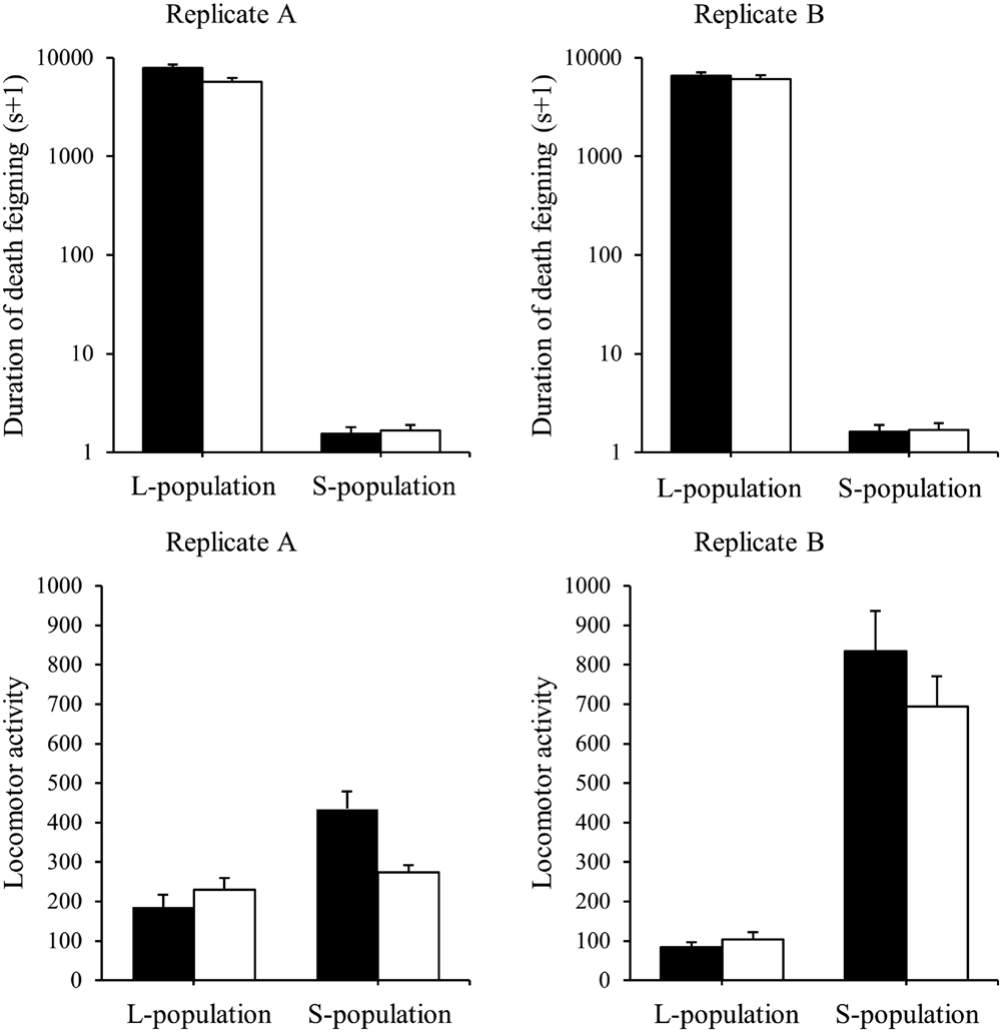
Duration of death-feigning and locomotor activity of *T. castaneum* as a function of bidirectional artificial selection on death-feigning behavior. L-populations selected for increased death-feigning, S-populations selected for short death-feigning. Black bars and white bars show male and female, respectively. Error bars show SE.

Populations selected for increased death feigning, feigned death for much longer than populations selected for reduced duration (Fig. 1, Table 1). There was a significant interaction between treatment and sex in the duration of death feigning (Table 1): females of the long-duration treatment tended to feign for less time than males, while the converse was true of the short-duration treatment. Adults from short-duration populations were also more active than beetles that feigned death for longer (Table 1). There was no difference in activity between sexes (Table 1), but there was again a treatment by sex interaction (Table 1): males tended to move less in the long-duration populations and more in the short.

**Table 1.** Results of GLMM for death-feigning and activity.

Trait	Factor	*d.f.*	*χ* ^2^	*P*
Death-feigning	Population	1	15510.32	< 0.0001
	Sex	1	1.16	0.2815
	Population × Sex	1	3.99	0.0458
	Error	394		
Activity	Population	1	60.17	< 0.0001
	Sex	1	2.39	0.1224
	Population × Sex	1	7.19	0.0073
	Error	224		

**Fig. 2.**
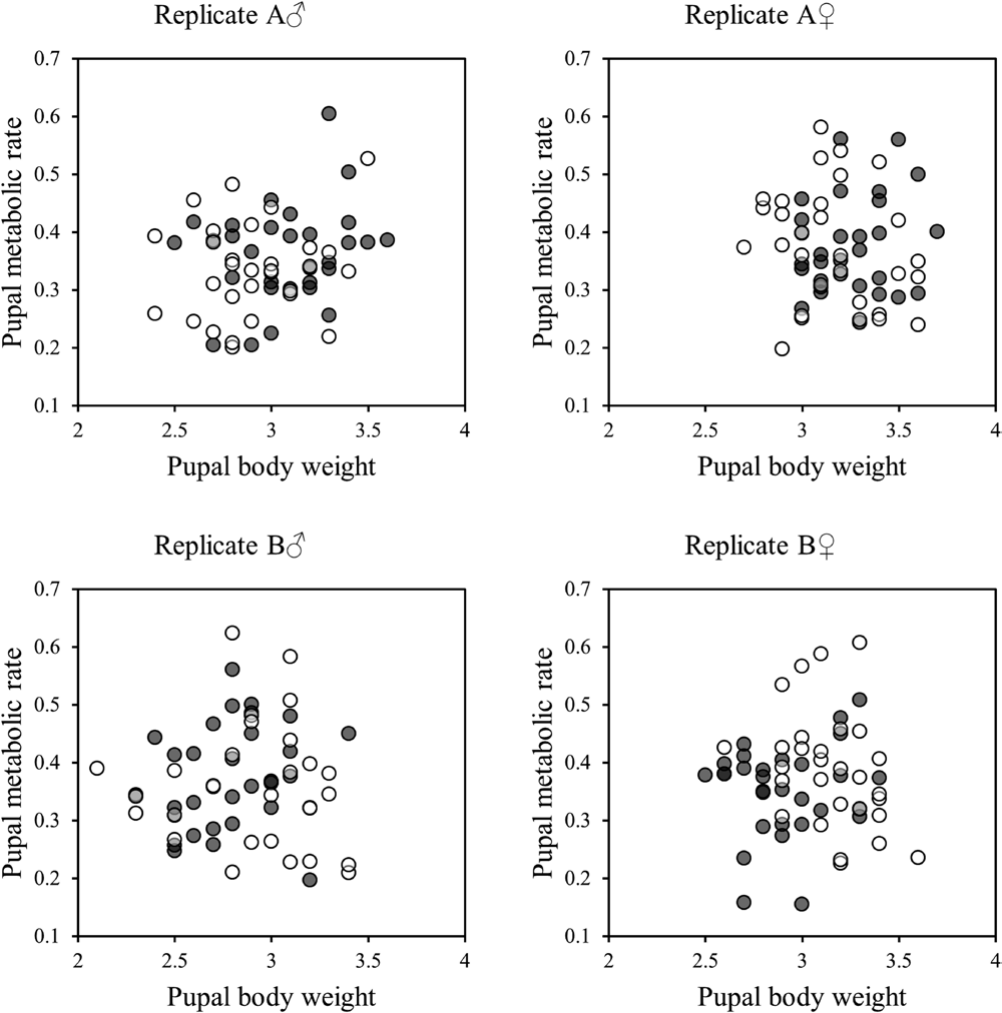
The association between body mass (mg) and metabolic rate (CO_2_ production: ml CO_2_/min × 1,000) at pupal stage of *T. castaneum*. Black and white circles show L and S populations, respectively.

As pupae, females were significantly heavier than males, but there was no other significant effects or interactions affecting pupal body mass (Table 2). There were no significant effects of treatment, sex and no interaction between treatment and sex affecting metabolic rate at the pupal stage (Fig. 2, Table 3), and nor was there a significant association between RMR and body mass in pupae (Table 3). Because of sexual size differences, we additionally regressed mass and MR separately for each sex, but again there was no significant associations (Table 4).

**Table 2.** Results of GLMM for body mass at pupa and adult, respectively.

Stage	Factor	*d.f.*	*χ* ^2^	*P*
Pupa	Population	1	0.39	0.5317
	Sex	1	32.24	< 0.0001
	Population × Sex	1	1.73	0.1879
	Error	232		
Adult	Population	1	0.03	0.8654
	Sex	1	7.80	0.0052
	Population × Sex	1	0.06	0.8026
	Error	204		

**Table 3.** Results of GLMM for RMR at pupa and adult, respectively.

Stage	Factor	*d.f.*	*χ* ^2^	*P*
Pupa	Population	1	0.07	0.7936
	Sex	1	1.02	0.3120
	Population × Sex	1	2.86	0.0907
	Body weight	1	0.20	0.6582
	Error	231		
Adult	Population	1	1.01	0.3161
	Sex	1	3.60	0.0577
	Population × Sex	1	0.99	0.3205
	Body weight	1	18.74	< 0.0001
	Error	203		

**Table 4.** Correlation between body mass and MR of males and females at pupal and adult stages, respectively.

Stage	Sex	Coefficient	*r* ^2^	*n*	*F*	*P*
Pupa	Male	0.0247	0.0068	119	0.81	0.3712
	Female	-0.0089	0.0006	119	0.08	0.7843
Adult	Male	0.3588	0.0872	100	9.37	0.0029
	Female	0.2794	0.1167	110	14.27	0.0003

**Fig. 3.**
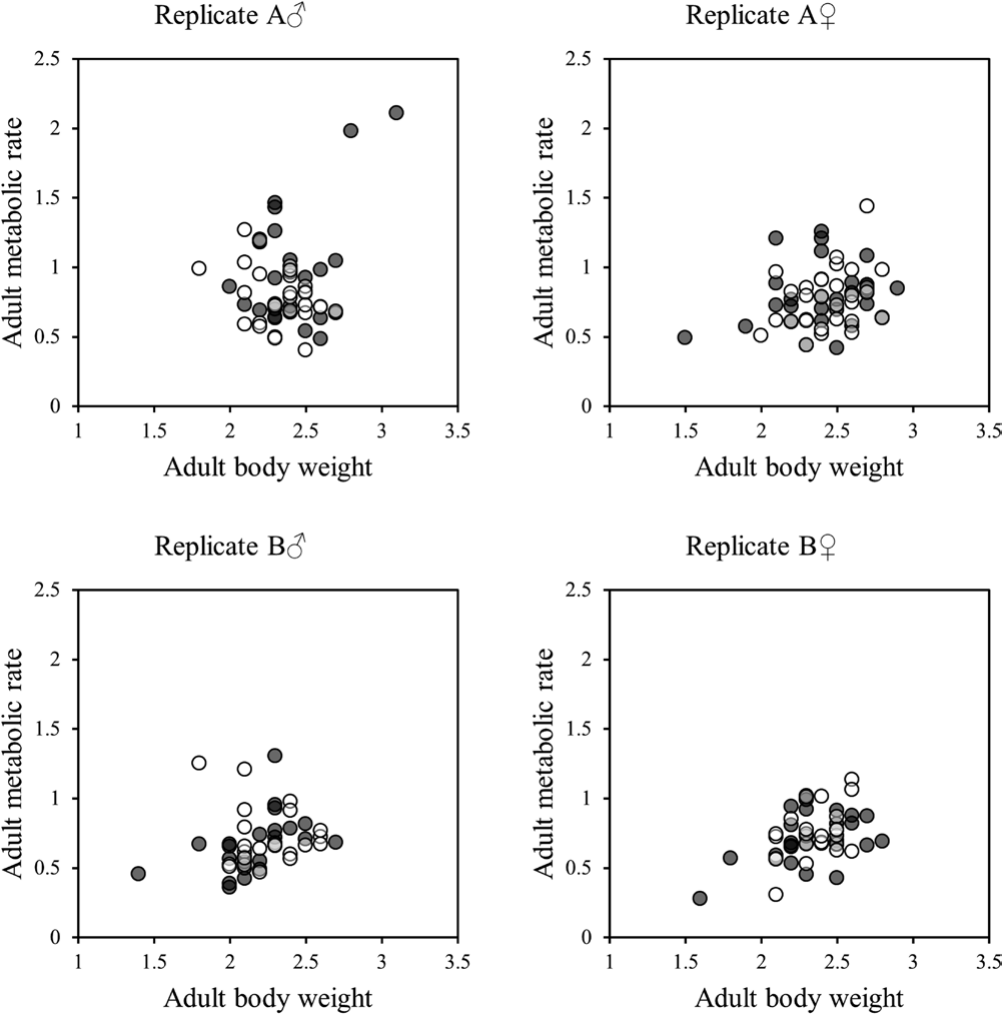
The association between body mass (mg) and metabolic rate (CO_2_ production: ml CO_2_/min × 1,000) at adult stage of *T. castaneum*. Black and white circles show L and S populations, respectively.

**Fig. 4.**
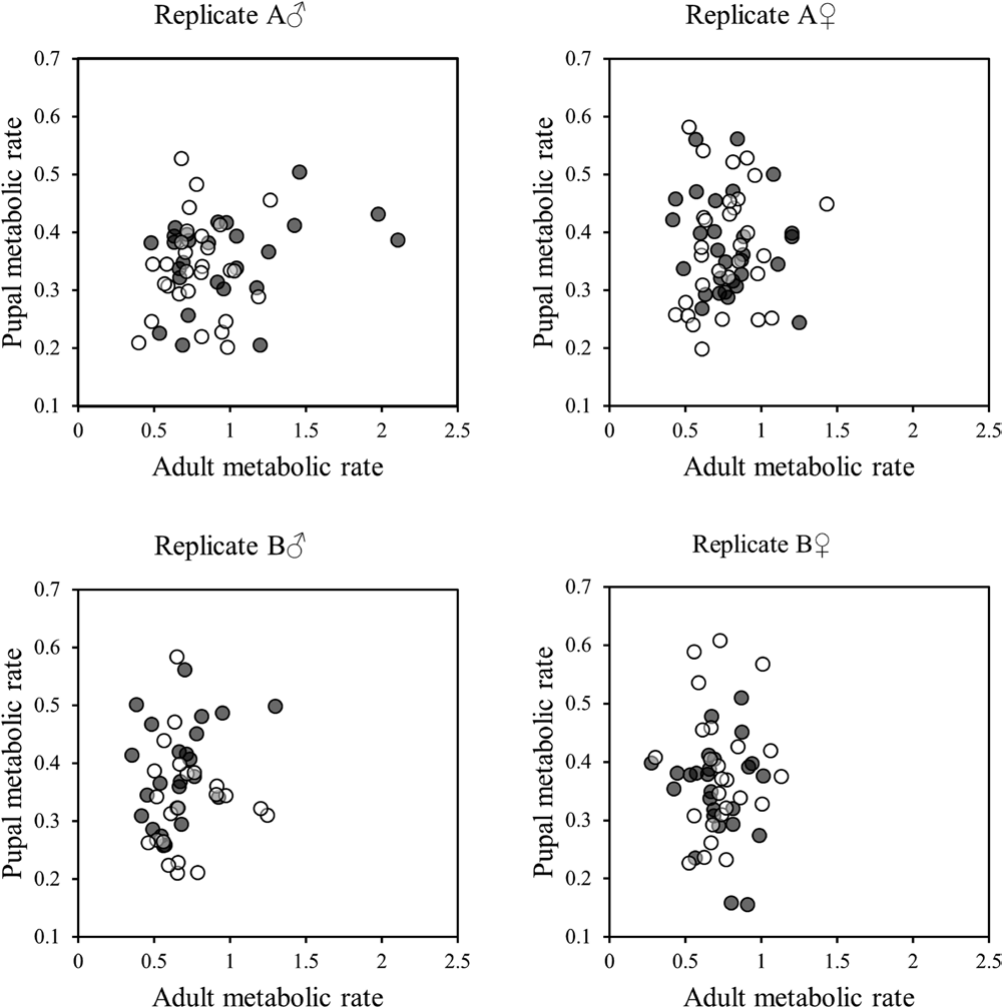
Correlation of metabolic rate (CO_2_ production: ml CO_2_/min x 1000) between pupal and adult stages of *T. castaneum*. Black and white circles show L and S populations, respectively.

As adults, females were again heavier than males (Table 2). However, there were no other significant effects or interactions affecting adult weight (Table 2). There were also no significant effects of treatment, sex, or their interaction on adult metabolic rate (Table 3). However, there was a significant association between body mass and MR at adult stage (Fig. 3, Table 3), and when split by sex, the association was stronger in females than males (Table 4). Finally, pupal body mass was positively correlated with adult mass (Table 5). However, there was no significant association between pupal and adult metabolic rate (Fig. 4, Table 5).

**Table 5.** Relationship between pupa and adult in body mass and RMR, respectively.

Trait	Coefficient	*r* ^2^	*N*	*F*	*P*
Body mass	0.4440	0.2776	210	79.91	< 0.0001
MR	0.2128	0.0058	210	1.22	0.2707

## Discussion

In this study, we used *T. castaneum* to test the hypothesis that duration of death feigning, which often associated with locomotor activity, is correlated with metabolic rate. However, there was no significant difference in metabolic rate between the L-populations with a longer death-feigning duration and the S-populations with a shorter death-feigning duration. This result suggests that duration of death feigning and metabolism are not genetically correlated in *T. castaneum*.

Selection on death feigning duration was successful as populations selected for increased thanatosis durations, feigned death for much longer than population selected for reduced durations, and as a correlated response to this selection, populations with reduced (increased) feigning had higher (lower) locomotor activity. This suggests that there is a negative genetic correlation between the duration of death feigning and locomotor activity, which accords with previous studies (eg Miyatake et al. 2008, Nakayama and Miyatake 2010, Matsumura et al. 2016, 2017). Moreover, this association persisted across multiple generations (under artificial selection) in these populations (Miyatake et al. 2008, Matsumura et al. 2017) suggesting either tight physical linkage or pleiotropy, rather than linkage disequilibrium for example (see Hosken and Wilson 2019). Given the negative correlation between death feigning and locomotor activity it seemed likely that individuals with longer death feigning would have lower RMR (Krams et al. 2013). However, no significant differences in RMR were found between selection regimes, either in pupae or adults. This result may be attributable to a variety of factors.

First, the relationship between metabolic rate and developmental period warrants consideration. Previous studies have reported that RMR is influenced by developmental period (Krams et al. 2017, 2018), and it is plausible that treatment specific effects on developmental period and locomotor activity counterbalanced each other. Unfortunately, we did not assess effect of developmental period on RMR but this is worthy of additional work.

Another potential reason for the lack of RMR effect is statistical power: we only had 2 replicate populations per treatment. However, we did detect difference in the duration of death feigning, the target trait of our artificial selection, and locomotor activity as a correlated response, and RMR was highly repeatable in adults. Additionally, physiological characters tend to have similar genetic variation to behavior (Mousseau and Roff 1987), and high repeatability shows the upper ceiling on RMR heritability is high (Boak 1989). In any case, since the effect of selection for death feigning on RMR is small, the results strongly suggested that there is no genetic correlation between death feigning and RMR in *T. castaneum*.

As with all negative results, it is also possible that our failure to detect a difference, results from measurement error, and although we did not directly assess this in pupae, the repeatability of MR in adults was high, but we note, population, rather than individual was our unit of replication. Additionally, the *r*^2^ value for our size-MR association in adults is within the *r*^2^ range size–MR associations from other insect studies, albeit at the lower end (eg Hack 1997, Nespolo et al. 2003, Van Voorhies et al. 2004a, Sinclair et al. 2011), which at least indicates our unexplained variation for this association is not too unusual. As a result, we do not think measurement error fully explains our negative result. Finally, the beetles used in this study had been subjected to bidirectional selection for 15 yr (see Matsumura and Miyatake 2018), and if anything, this should reduce within-individual variation within populations thereby maximizing our ability to detect treatment differences. However, we detected no treatment effects on MR.

Negative phenotypic associations between the duration of death feigning and RMR have been documented in the yellow mealworm *T. molitor* (Krams et al. 2013). Aside from inherent species difference, the contrast between our findings and those with the mealworm are highlighted by mean CO^2^ production differences between the 2 species: the average metabolic rate of *T. molitor* adults is about 0.82 (± 0.68) VCO^2^ μl/min (mean ± SE), whereas in *T. castaneum* it was much lower at 0.05 (± 0.001) μl/min. Thus *T. molitor* has a much higher MR (approximately 17 times) than *T. castaneum*, even considering body size differences—*T. molitor* is larger than *T. castaneum*.

Studies report correlations between RMR and activity that are positive, negative and non-existent (Careau et al. 2011, Le Galliard et al. 2013). Our work is consistent with the latter and a finding that walking activity was not correlated with MR in *T. castaneum* (Arnold et al. 2017). It would be interesting to examine possible associations between death feigning behavior and other measures of metabolism (eg maximum metabolic rate or daily energy expenditure: Careau et al. 2008, White et al. 2016). In any case, if the death feigning/locomotion association can be thought of as a behavioral syndrome (sensu Dall et al. 2004, Careau et al. 2008), and we did not find any clear link between personality and energy expenditure.

It has been suggested that personality differences may be linked with MR because consistent differences in traits like movement or stress could impact metabolism (Careau et al. 2008). Although responses to artificial stimuli, as used here, tend to reflect natural responses (Careau et al. 2008), there is debate about how to best measure MR in testing for links between MR and personality. Be that as it may, like us, other studies have also failed to find clear associations (e.g., White et al. 2016). Despite our failure to find associations here, we suggest that divergent selection to generate very large differences in behavior should be a good way to test for possible personality-MR links.

Despite finding no death-feigning–MR associations, correlations between MR and size in adults were detected. Body size–metabolic rate associations are predicted and found across taxa, with more than 90% of the inter-specific variation in MR explained by mass in birds and mammals (McNab 2002: also see, Withers 1992). Intra-specific associations are generally much weaker, and in insects, associations between mass/size and metabolic rate associations are frequently (e.g., Lighton 2008, Okada et al. 2011, Sinclair et al. 2011) but not always detected (van Voorhies et al. 2004b; Canzano et al. 2006, Oikawa et al. 2006). It has been suggested that failure to find metabolic rate/size associations in some insect investigations may reflect differences in time in captivity and starvation resistance (eg Harshman et al. 1999; Van Voorhies et al. 2004a), but cyto-nuclear epistasis can also affect MR (Arnqvist et al. 2010), as can development stage (Freitak et al. 2003). We failed to find a mass-MR association at the pupal stage, which could relate to variation in development rate (eg smaller pupae developing faster, and in some insects development rate is highly plastic (Blanckenhorn 1997), but unfortunately we did not assess development rate. Additionally, although body mass was positively associated between pupal and adult stages, metabolic rate was not correlated across the 2 life history stages, consistent with developmental stage specific metabolism (Freitak et al. 2003).

In conclusion, this study provides evidence that selection for death-feigning behavior elicits a correlated response in locomotor activity levels. However, it does not appear to induce a correlated response in resting metabolic rate. While these findings contribute to the existing body of evidence regarding the relationship between death feigning, locomotor activity and resting metabolic rate, they also indicate that further research across taxa is necessary to elucidate this aspect in greater detail.

## Supplementary Material

ieaf007_suppl_Supplementary_Figures_S1
