# Supplementary material for: Behavior, behavioral syndromes, and metabolism: the effects of artificial selection for death-feigning on metabolic rate
Source: J Insect Sci. 2025 Feb 15;25(1):16. doi: 10.1093/jisesa/ieaf007 (PMC11829217; doi:10.1093/jisesa/ieaf007)
Supplement: ieaf007_suppl_Supplementary_Figures_S1 [file ieaf007_suppl_supplementary_figures_s1.docx]

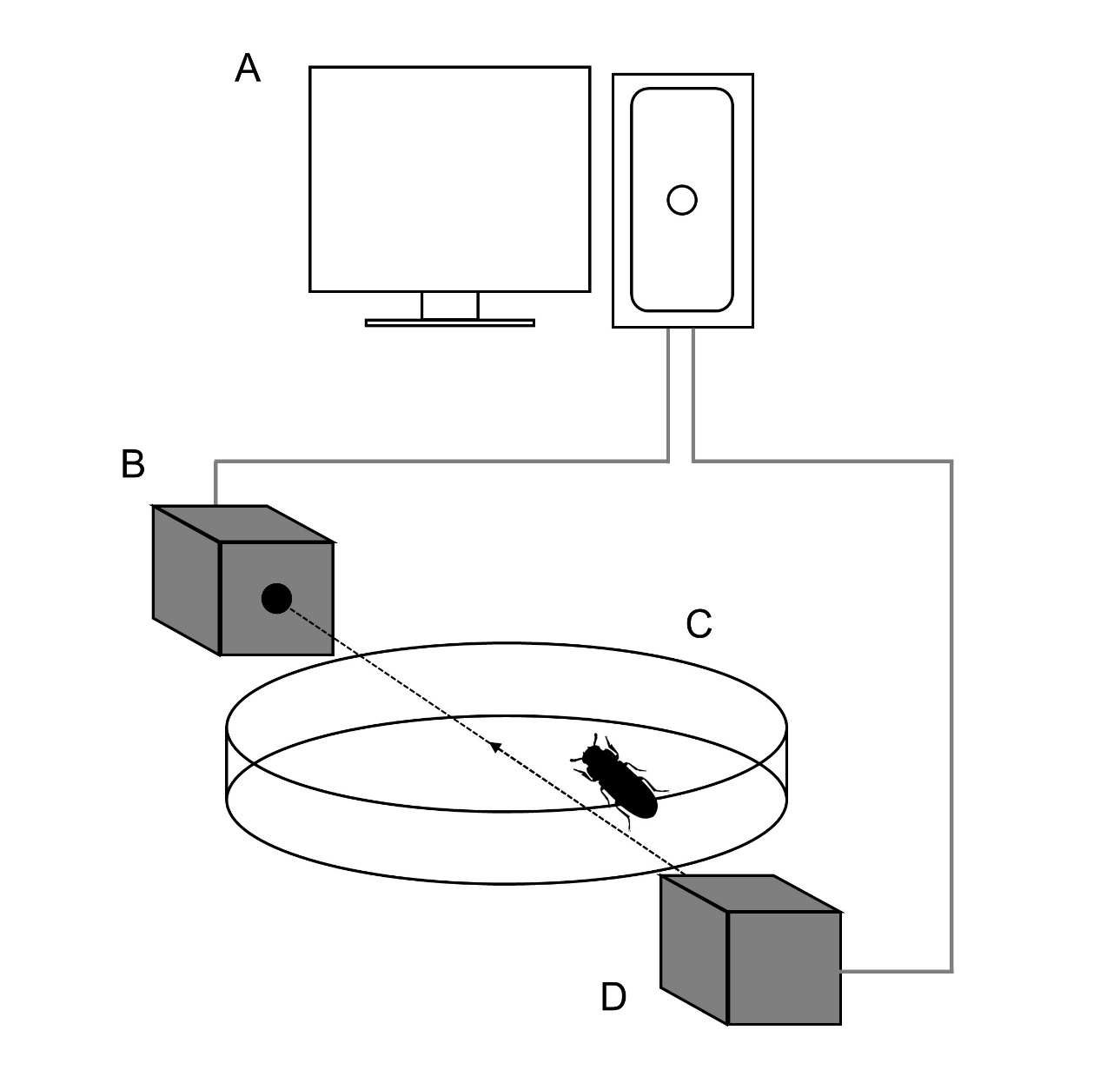


**Figure S1**. Infrared actograph system. In this monitoring system, an infrared light beam was passed between emitter (B) and detector (D) (E3S-AT11; Omron, Kyoto, Japan) through a Petri dish (C) that included a beetle without food. When the beetle interrupted the light beam, a signal is recorded immediately in PC (A). In this measurement, locomotor activity was defined as the number of interruptions of the light beam in 24h.
